# Impact of dynamic co-evaporation schemes on the growth of methylammonium lead iodide absorbers for inverted solar cells

**DOI:** 10.1038/s41598-022-23132-w

**Published:** 2022-11-10

**Authors:** Robert Heidrich, Karl L. Heinze, Sebastian Berwig, Jie Ge, Roland Scheer, Paul Pistor

**Affiliations:** 1 Martin-Luther-University, Von-Danckelmann-Platz 3, 06120 Halle (Saale), Germany; 2 Universidad Pablo de Olavide, c/ Utrera 1, 41013 Sevilla, Spain; 3 Fraunhofer CSP, Otto-Eißfeldt-Straße 12, 06120 Halle (Saale), Germany

**Keywords:** Devices for energy harvesting, Energy science and technology, Renewable energy, Solar energy, Solar cells, Electronic devices, Materials science, Electronic properties and materials, Semiconductors, Physics, Electronic and spintronic devices

## Abstract

A variety of different synthesis methods for the fabrication of solar cell absorbers based on the lead halide perovskite methylammonium lead iodide (MAPbI_3_, MAPI) have been successfully developed in the past. In this work, we elaborate upon vacuum-based dual source co-evaporation as an industrially attractive processing technology. We present non-stationary processing schemes and concentrate on details of co-evaporation schemes where we intentionally delay the start/end points of one of the two evaporated components (MAI and PbI_2_). Previously, it was found for solar cells based on a regular n-i-p structure, that the pre-evaporation of PbI$_2$ is highly beneficial for absorber growth and solar cell performance. Here, we apply similar non-stationary processing schemes with pre/post-deposition sequences for the growth of MAPI absorbers in an inverted p-i-n solar cell architecture. Solar cell parameters as well as details of the absorber growth are compared for a set of different evaporation schemes. Contrary to our preliminary assumptions, we find the pre-evaporation of PbI_2_ to be detrimental in the inverted configuration, indicating that the beneficial effect of the seed layers originates from interface properties related to improved charge carrier transport and extraction across this interface rather than being related to an improved absorber growth. This is further evidenced by a performance improvement of inverted solar cell devices with pre-evaporated MAI and post-deposited PbI_2_ layers. Finally, we provide two hypothetical electronic models that might cause the observed effects.

## Introduction

Lead halide perovskite semiconductors have excelled in recent years as versatile semiconductors in a variety of opto-electronic applications^1,2^. Most prominently, laboratory scale solar cells, both in single junction (η > 25 %) and tandem configuration with Si (η > 29.5%), have shown rapidly increasing record efficiencies well beyond expectations^3^.

Apart from device stability, main concerns in view of an industrial uptake of the technology are the scalability and reproducibility of the implemented fabrication processes. While many technological advances on small, laboratory scale solar cells have been obtained with wet-chemical methods (e.g. spin-coating, printing), some vacuum-based approaches have also been successfully implemented.

Liu et al. reported the fabrication of efficient, planar perovskite solar cells by dual source co-evaporation, using methylammonium (MA) and PbCl$_2$ as precursors reaching efficiencies above 15%^4^. Several other groups have followed this route (with either PbI$_2$ or PbCl$_2$ as lead halide precursors)^5^ and in 2019 the use of optimized contact layers by Bolink et al. led to efficiencies exceeding 20%^6^. Co-evaporation generally leads to compact, homogeneous films, is fast and easily scalable and offers an improved processing control under reproducible conditions. The two main approaches used nowadays are based either on a) simultaneous, stationary co-evaporation from different sources or b) sequential processing, where first only one component is deposited (normally the lead halide, e.g. PbI$_2$). This precursor layer is then converted into the perovskite, e.g. through exposure to an MAI atmosphere or by deposition of the MAI followed by an annealing^7,8^.

Attempts to partly combine the two approaches are rare, i.e. to move to non-stationary co-evaporation where the application of the two precursors is not completely synchronized and stationary. This is even more astonishing, as this is in fact one of the main advantages of co-evaporation in comparison to solution-based processing: the amount and ratio of precursors arriving at the substrate can be varied during processing. As an example for other photovoltaic technologies, high efficiency co-evaporated solar cells based on chalcopyrite Cu(In,Ga)Se$_2$ absorbers are prepared with a complex Cu-poor/Cu-rich/Cu-poor evaporation scheme leading to optimal absorber properties and a carefully designed band gap gradient within the absorber^9^.

In this sense, non-stationary co-evaporation not only bears the potential to vary the composition of the absorber during growth, but also to initiate or terminate the growth with specific precursor compositions. Furthermore, it is rather difficult in an industrial in-line fabrication, where the substrates are normally transported across a series of linear evaporation sources, to ensure the continuously homogeneous, stationary flux of constant precursor ratios to the substrate, as is the case in a stationary laboratory setup.

These considerations motivated us to investigate the impact of pre-evaporating of precursors, starting the evaporation with seed layers instead of a continuous evaporation. This approach continues our previous work in this direction for solar cells in a regular n-i-p configuration^10^. To the best of our knowledge, this is so far the only work considering such kind of asynchronous, non-stationary co-evaporation, where we found that a pre-evaporation of PbI$_2$ seed layers was greatly beneficial to the solar cell performance. We argumented, that the PbI$_2$ seed layer initiated crystallization, increased the sticking coefficient and led to the growth of a MAPI absorber with improved properties. However, ultimately it remained unclear whether the performance improvement originated from an improved absorber growth (as a result of the different nucleation on the PbI$_2$ seed layer) or due to an improved interface to the electron transport layer (ETL).

In order to elucidate this question, here, we present our first results on novel pre/post-evaporation schemes for inverted perovskite solar cells based on a p-i-n structure with NiO as hole transport layer (HTL) and an ETL double layer based on [6,6]-Phenyl C61 butyric acid methyl ester (PCBM) and ZnO. We use different pre/post-deposition schemes of MAI and PbI$_2$ and report their impact on absorber growth in terms of crystallization and morphology and on performance in complete solar cell devices.

The motivation to use an inverted solar cell configuration is two-fold: On the one hand, it allows us to directly compare the results obtained in our previous work and study the impact of pre/post-evaporation schemes for an inverted contact layer configuration (PbI$_2$ pre-evaporation onto the HTL instead of the ETL layer). On the other hand, the use of an inverted device configuration is motivated by our longterm goal to fabricate tandem devices, where a solar cell in p-i-n configuration is needed to ensure the correct direction of the current flow in the top cell with respect to the diode of the bottom cell (p-type Si or chalcopyrite solar cell).

## Experimental methodology

Solar cells were prepared on glass substrates coated with transparent conductive indium-tin-oxide (ITO) layers in an inverted p-i-n structure. The used stacked cell architecture is based on NiO as hole transport layer (HTL) and a bilayered electron transport layer (ETL) made of phenyl-C61-butyric acid methyl ester (PCBM) and ZnO nanoparticles. The complete solar cell consists of a glass/ITO/NiO/MAPI/PCBM/ZnO/Ag layer stack. Details concerning the sample preparation, ETL/HTL deposition parameters, the solar cell fabrication and characterization methodology can be found in the supporting information.

The co-evaporation of MAPI absorbers was carried out in a high vacuum chamber (base pressure $10^{-5}$mbar) with two evaporation sources filled with MAI and PbI$_2$, respectively. The setup is described in detail in the work by Heinze et al., where dynamic processing schemes including the pre-deposition of different seed layers were developed for regular solar cells in n-i-p structure in the same system^10^. Further experimental details on the evaporation setup and the characterization methodologies can be found in the supporting information.

The MAPI absorbers were synthesized by co-evaporating MAI and PbI$_2$. Optimal deposition parameters concerning the optimal flux ratios for near stoichiometric absorber compositions had been determined previously (see reference^10^). Following this work, we ramped both crucibles to their respective target temperature in 900s. A constant PbI$_2$ source temperature of 288$^\circ$C was used for all experiments. The target temperature for the temperature ramp of the MAI crucible was set to 115$^\circ$C. Due to its high vapor pressure and particle scattering, MAI does not evaporate directionally^5^. As a consequence, the MAI flux cannot be easily controlled by the source temperature alone, nor the installed quartz crystal microbalance. In accordance with^10,11^, the MAI flux was therefore controlled by adjusting the MAI source temperature in order to maintain a constant working pressure within the evaporation system. The optimal constant working pressure had been previously determined and was set to 7.5$\times 10^{-5}$mbar^10^.

Our evaporation setup is equipped with an in situ X-ray diffraction setup (in situ XRD). It consists of a Cu K$\alpha$ X-ray source and linear detector array arranged at opposite sides of the vacuum chamber. The X-rays enter and leave the chamber through Kapton windows allowing the recording of XRD scans in an angular 2$\Theta$-range of 28${^\circ }$. Details on the experimental setup can be found in the supporting information and in references^10,12^.

**Figure 1:**
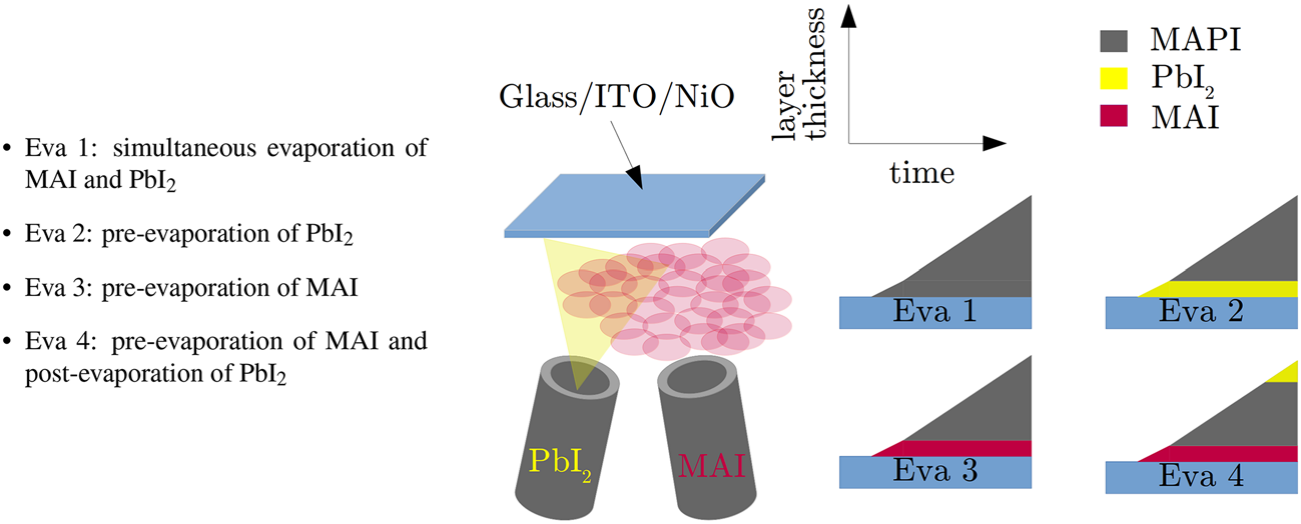
Schematic representation of the co-evaporation schemes using PbI$_2$ and MAI precursors on glass/ITO/NiO substrates to grow MAPI. While PbI$_2$ evaporates as a molecular stream, MAI is distributed homogeneously in the chamber, increasing the total chamber pressure^13,14^. Different evaporation schemes (Evaporation 1-4) by choice of different initial/final evaporation conditions have been tested and compared with regard to their impact on the crystal growth and the electronic properties of the absorber.

In this work, MAPI absorbers were deposited on glass/ITO/NiO substrates in a variation of four different evaporation schemes. Every process was carried out with at least 4 samples (3 solar cells on each sample) allowing a small statistical comparison. A nitrogen filled glovebox is directly attached to our evaporation system, allowing the sample/source material insertion/extraction under inert working conditions. The four different evaporation schemes are schematically illustrated in Fig. 1 and will be named throughout this work with the following abbreviations.

The entire evaporation time was 10100s for Eva 1-3 and 10700s for Eva 4. Pre- or post-evaporation times were set to 600s leading to total absorber thicknesses in the range of 300nm. According to our previously performed flux measurements, the layer thickness during the pre/post-evaporation sequences of 600s is in the range of 10nm to 20nm.

After the MAPI evaporation was finished, 3 out of 4 samples were further processed to solar cells in a nitrogen atmosphere by adding a PCBM/ZnO electron transport layer (ETL). Finally, Ag contacts were evaporated in a separate vacuum chamber. j-V characteristics of the solar cells were measured under simulated AM1.5 illumination. Details on the sample preparation and contact layer deposition can be found in the supporting information. On the remaining sample, TRPL, SEM and EDX measurements were conducted in this order. For SEM analysis, the sample was cut in half allowing cross-sectional imaging.

## Results

### Evaporation process

**Figure 2:**
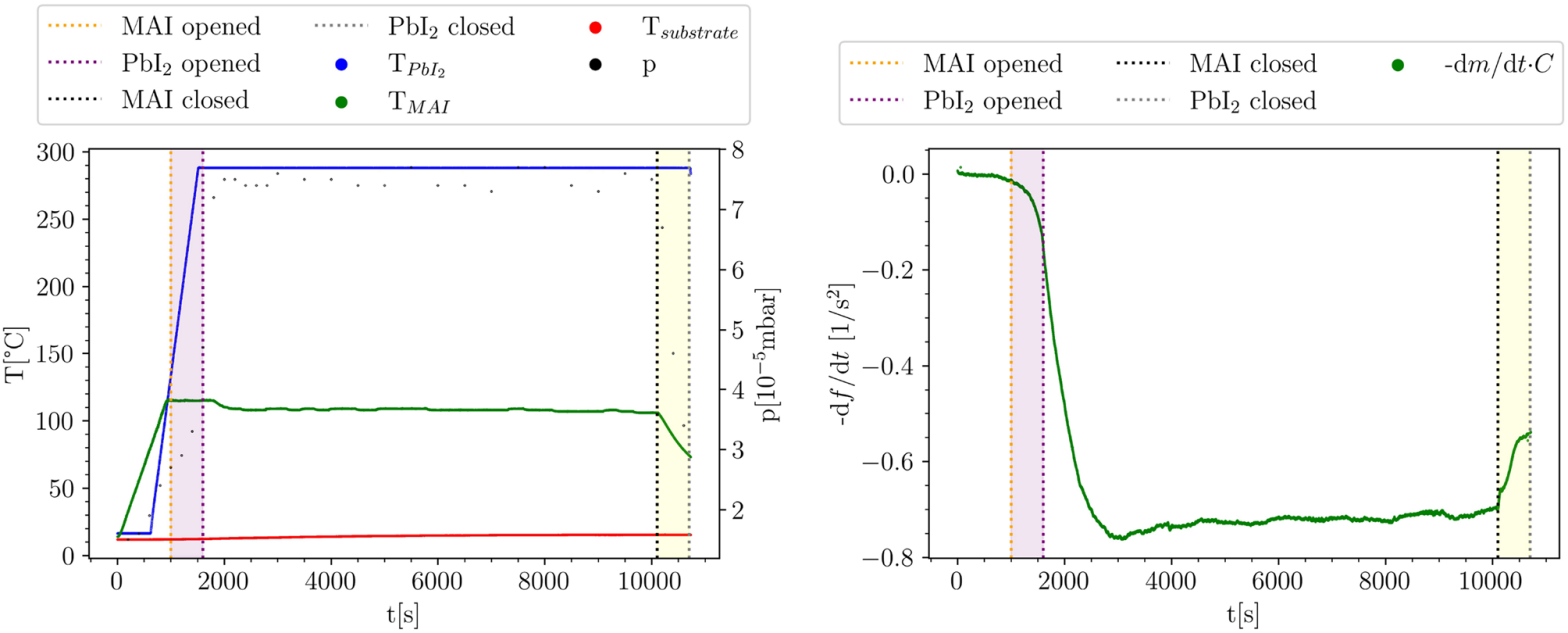
Pre-evaporation of MAI in combination with post-deposition of PbI$_2$ (Eva 4). The left plot shows the temperatures and pressure for the whole evaporation time, the right image visualizes the time derivative of the frequency calculated from the measured frequency by the QCM.

Figure 2 shows EVA 4 as an example for the evolution of the crucible temperatures, chamber pressure (left) and the quartz crystal micro balance (QCM) reading for the evaluation of the deposited mass (right). A comparison of the remaining evaporation schemes can be found in the supporting information. The shutter control for the pre-deposition of MAI and post-deposition of PbI$_2$ was adjusted to the heating scheme as indicated with dashed lines in the plots. Pre- and post-deposition intervals are marked by the colored rectangles (purple: MAI pre-evaporation, yellow: PbI$_2$ post-evaporation). For the pre-deposition of MAI, the MAI deposition was started 600s earlier and accordingly, the post-deposition of PbI$_2$ in Eva 4 was carried out by turning off the MAI heater, closing the MAI shutter and continuing with the deposition of PbI$_2$. The substrates were not actively heated and the substrate temperature stayed approximately constant for all evaporations starting at $T_{subs} =$ 12 °C and reaching $T_{subs} =$ 16 °C at the end of the process.

At the right hand side of Fig. 2, the time derivative of the QCM frequency is displayed. This quantity is proportional to the rate of deposited mass modified by material constants of the quartz crystal and visualizes the deposition kinetics^15^. The development of the time derivative of the QCM frequency shows that some mass is already deposited before the shutters were opened, which is explained by the non-directional evaporation behaviour of MAI^13,14^. After the MAI shutter was opened, the slope increases, in accordance with the observed increase in chamber pressure (left). When the PbI$_2$ shutter is opened, the slope increases again until a stable deposition is reached at approximately 3000s after the process was started. When the MAI shutter is closed the deposition rate decreases slightly due to the missing MAI flux indicating the post-deposition of PbI$_2$.

**Figure 3:**
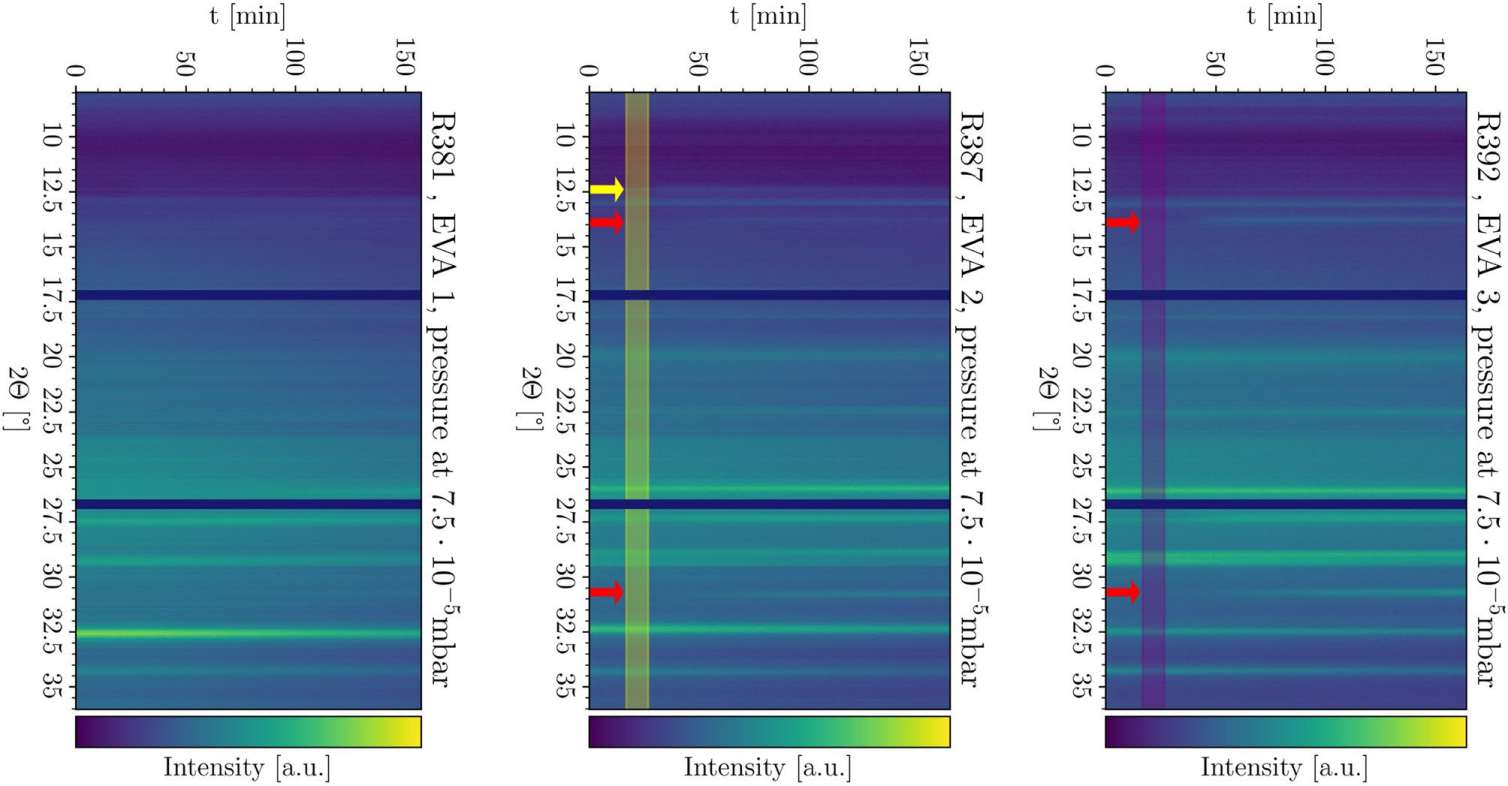
In situ XRD scans of Eva 1 (left), Eva 2 (middle) and Eva 3 (right). The yellow arrow marks the significant PbI$_2$ peak while the red arrows indicate the significant MAPI peaks^16,17^. Pre-evaporation intervals are marked by colored rectangles (yellow: PbI$_2$ pre-evaporation, purple: MAI pre-evaporation).

The deposition processes were monitored with the in situ XRD system attached to the evaporation chamber as displayed in Fig. 3. Here, the evolution of the XRD intensity is color-coded in color maps where the x-axis represents the evolution of process time and the y-axis represents the diffraction angle. The color maps are normalized to the maximum value of each measurement. All peaks which are visible from the beginning either belong to the substrate (glass/ITO/NiO) or the substrate carrier and will not be discussed here. The detector assembly has two blind spots at 17.5${^\circ }$ and 26.5${^\circ }$.

Eva 1 did not show any signs of crystallization or crystalline thin film growth; no peaks correlated to MAPI, MAI or PbI$_2$ were observable. However, visually, the substrates were dark after the co-evaporation process and SEM cross-sections confirmed deposition of a thin film of 300nm. For the case of Eva 2, a PbI$_2$ (001) peak at 12.5${^\circ }$^16^ can be observed starting approximately 30min after the beginning of the process. After approximately 60min, tetragonal MAPI (110) and (114) peaks^17^ at 14${^\circ }$ and 31.5${^\circ }$, respectively, become visible. For the third evaporation scheme with MAI pre-evaporation (EVA 3), no PbI$_2$ peak was detected. The tetragonal MAPI peaks appeared earlier at approximately 45min and with increased intensity relative to the substrate peaks.

### SEM and EDX measurements

**Figure 4:**
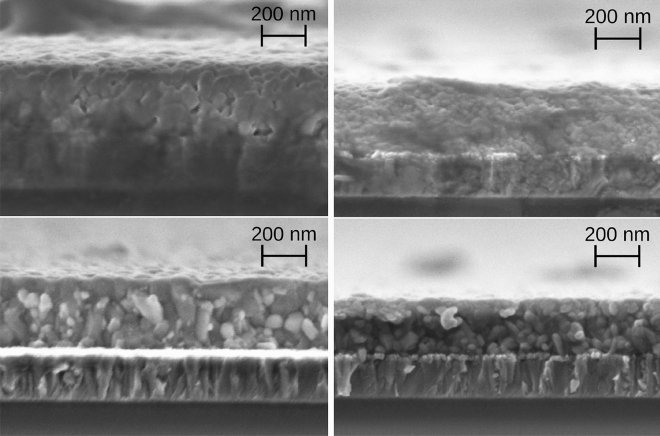
Cross-sectional SEM images of the perovskite absorbers of Eva 1 (top left), Eva 2 (top right), Eva 3 (bottom left) and Eva 4 (bottom right). The magnification was fixed at 80000.

Figure 4 displays cross-sectional scanning electron microscope (SEM) images of the absorbers. The absorber thickness of Eva 1-3 was measured to be approximately 300nm, only Eva 4 was slightly thinner at approximately 250nm. In between the absorber and the ITO layer (ca. 180nm), the NiO layer can be identified as a slim, bright line with distinct grain structure and a thickness of approximately 25nm. The morphology of the EVA 1 absorber in Fig. 4 shows round particles and some porosity, while for the absorber of Eva 2 no distinct granular structure is observed, but a rather homogeneous cross-section. For Eva 3 and Eva 4, clearly distinguishable granular structures of few tens to one hundred nanometers can be distinguished. Further discussion and morphology images can be found in the supporting information.

Detecting lighter elements like carbon or nitrogen was not possible with sufficient accuracy in the used setup^18^, impeding the direct quantification of methylammonium via EDX. In Table 1 the results of the EDX measurements for Pb and I are listed for comparison of all processes. The stoichiometric perovskite has the chemical formula ABX$_3$^19^ leading to a nominal $\frac{I}{Pb}$ ratio of 3. We evaluate the stoichiometry according to the [I] to [Pb] ratio $\frac{I}{Pb}$, where values above 3 would be expected for MAI-rich absorbers and values below 3 indicate PbI$_2$-rich perovskites. We conclude that all absorbers had a near stoichiometric composition, with Eva 1, 2 and 4 being slightly PbI$_2$-rich, while Eva 3 (with pre-deposited MAI) was slightly MAI-rich (I/Pb ratio of 3.08).

**Table 1:** EDX measurement of the absorbers from different evaporation processes. The atomic fractions were normalized to 100%. A relative error of 4% for all measurements was considered as reasonable and calculated by the EDX software.

	Eva 1	Eva 2	Eva 3	Eva 4
I atom [%]	73.47	74.03	75.49	74.23
Pb atom [%]	26.53	25.97	24.51	25.77
$\frac{I}{Pb}$	2.77	2.85	3.08	2.88

### TRPL measurements

**Figure 5:**
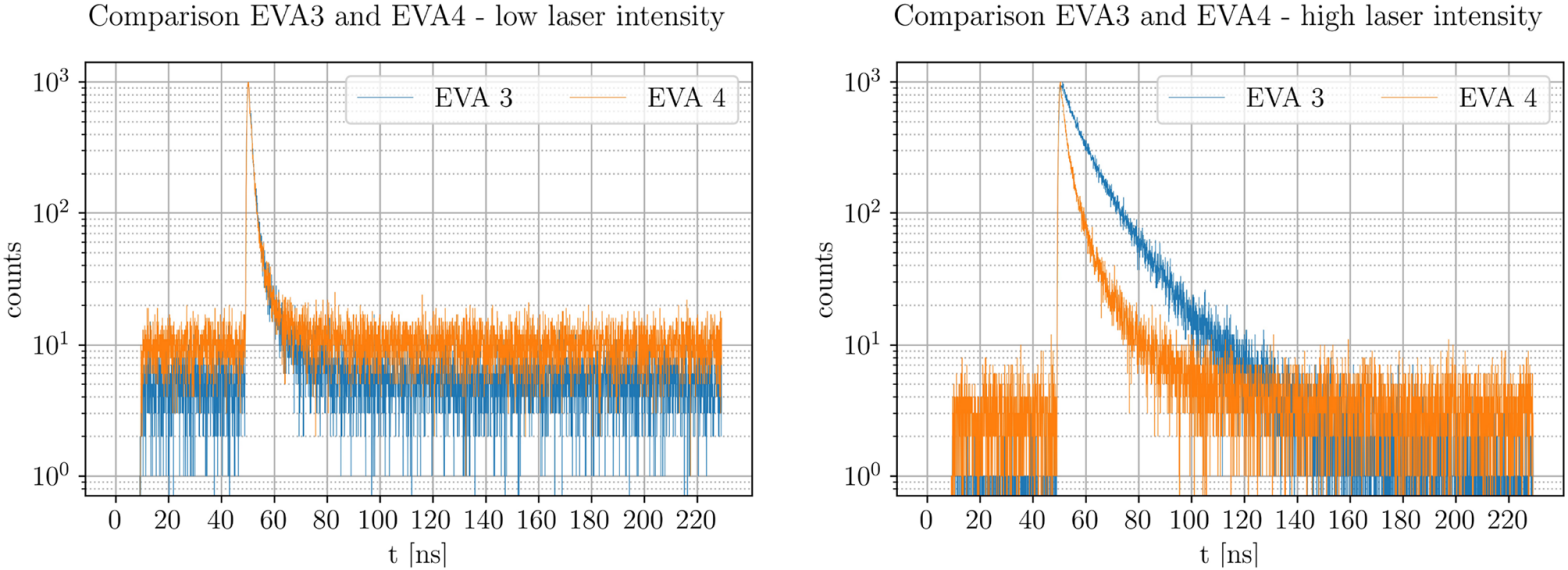
Comparison of TRPL measurements of Eva 3 and Eva 4 with low laser intensity ($0,001 \cdot I_0$, left) and high laser intensity ($I_0$, right). The incident beam was focused on the absorber top side, which was also the area of photon detection. Attenuation of the laser intensity to $0,001 \cdot I_0$ was made possible by using a neutral gray filter.

The influence of a post-deposited PbI$_2$ top layer (Eva 4) was analyzed qualitatively by TRPL measurements of the absorber from the top side. Figure 5 only displays the lowest ($0,001 \cdot I_0$) and highest laser ($I_0$) intensities that were used in a series of different intensities. We did not detect any photo degradation during these measurements and during subsequent control measurements.

In general, the observed photoluminescence decays are not strictly mono-exponential, indicating a combination of recombination processes^20^. The decay times for the low intensity curves are approximately similar for both evaporation schemes. A built-in electric field rapidly separating the generated charge carriers is one possible explanation for the short decay times at low injection levels, an effect that would be counterbalanced by the generated photovoltage at higher injection levels. At high illumination conditions, the measured signal is significantly different between Eva 3 and Eva 4. The faster decay of Eva 4 could be explained by improved charge carrier extraction due to the post-evaporated lead iodide layer. This would result in less charge carrier accumulation and therefore quenching of the TRPL signal.

### j-V analysis

**Figure 6:**
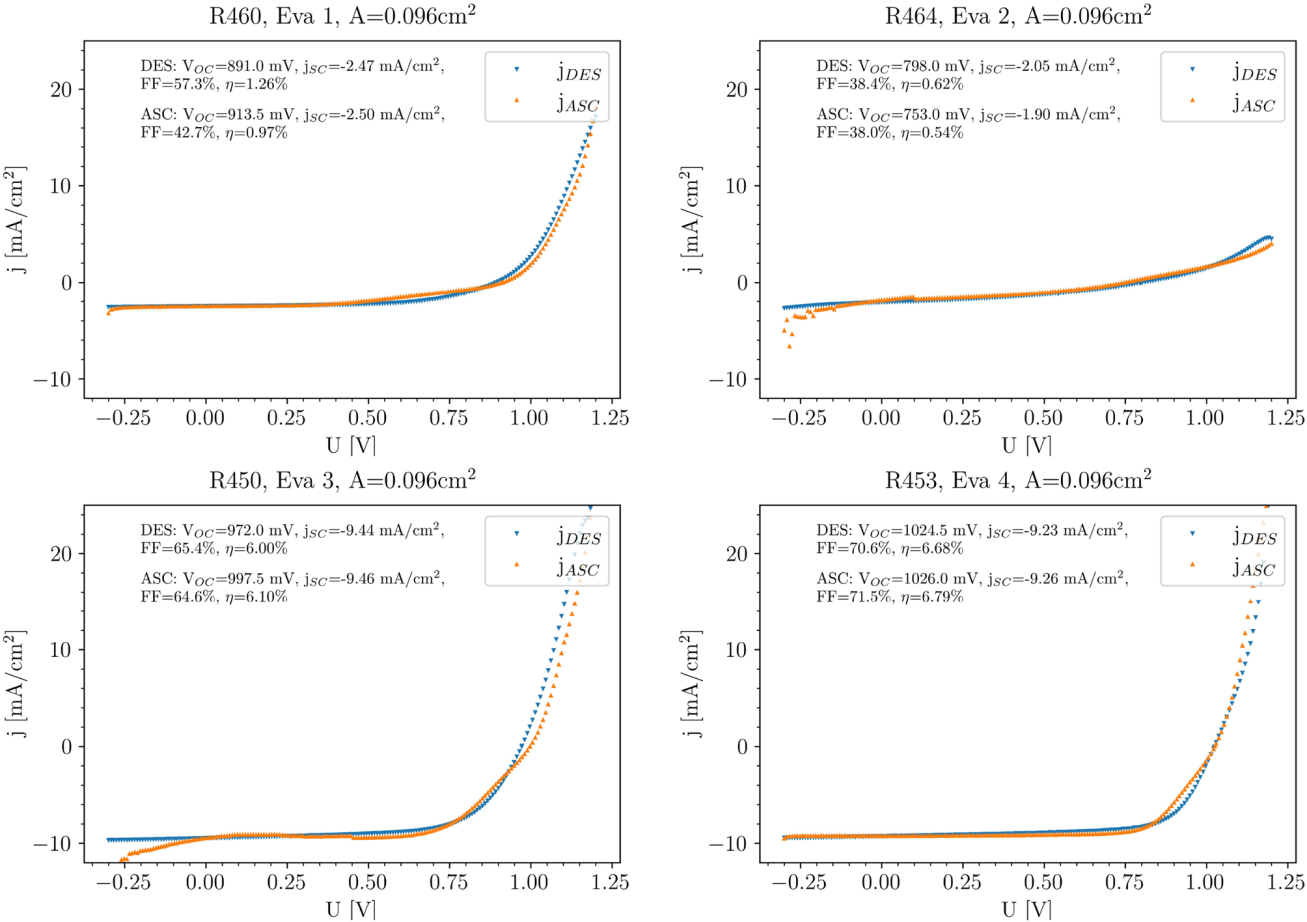
j-V characterisation of Eva 1 (top left), Eva 2 (top right), Eva 3 (bottom left) and Eva 4 (bottom right).

After completing the solar cell devices by deposition of ETL and contact layers, j-V curves were measured under simulated AM1.5 sun light in ascending and descending voltage sweep directions. The j-V curves of the best performing solar cells measured in ascending and descending direction are plotted for each evaporation run exemplary in Fig. 6, while averaged solar cell parameters are listed in Table 2. A more detailed analysis of the data distribution of the solar cell parameters for each evaporation scheme can be found in the supporting information.

The best solar cell for the simultaneous (no pre-/post-deposition) evaporation scheme (Eva 1, top left graph in Fig. 6) resulted in a device with rectifying behaviour, an open circuit voltage around 900mV, low fill factor (42 % in ascending and 57 % in descending direction) and little hysteresis. Due to the rather low short circuit current density of less than 3mA/cm$^{2}$, the efficiency of the best device for this evaporation scheme was limited to approximately 1 %. When PbI$_2$ is pre-evaporated (EVA 2, top right graph), the performance of the corresponding solar cells is even lower, mainly because of the drastically decreased fill factor (38% in both directions). The j-V curve now nearly shows no rectifying behaviour and the average efficiency of all solar cells from this evaporation scheme was limited to well below 1 %. This stands in clear contrast to our expectations and the results from Heinze et al. in^10^, where the PbI$_2$ pre-evaporation had led to an increased short circuit current density and overall performance in n-i-p based solar cells.

On the contrary, a large improvement in the short circuit current density was observed for EVA 3 (bottom left), were MAI instead of PbI$_2$ was pre-evaporated. For this evaporation scheme, solar cell efficiencies exceeding 6 % were obtained. A further improvement was observed by additional post-deposition of PbI$_2$ (Eva 4, bottom right): here, the efficiency of the best solar cell is elevated to 6.7 % (descending) and 6.8% (ascending). This increase is mainly due to the better open circuit voltage exceeding 1V and an improved fill factor (above 70 %).

Overall solar cell efficiencies of the devices presented in this work are well below the current state-of-the-art of evaporated perovskite solar cells. Despite this, a clear impact of the evaporation scheme on the solar cell performance can be observed in both the j-V curves of the best cells and the list of averaged parameters. We would like to stress the fact that we have reproduced these results and that the co-evaporation methodology and equipment employed here are identical to the ones presented in our previous publication (^10^) and therefore have proven to provide absorber-grade MAPI with reasonable efficiencies. Despite our efforts, in the inverted p-i-n configuration we have not been able so far to produce solar cells with co-evaporated MAPI exceeding 8% efficiency, in contrast to the regular n-i-p configuration where we achieved efficiencies around 15%. As the same processing conditions have been applied, we believe that the main problems of our p-i-n devices lay still in the contacting layers, which demand further optimization.

**Table 2:** Average and standard deviation (SD) of the solar cell parameters corresponding to the different evaporation schemes. The total amount of measured solar cells and the parameter distribution for the corresponding statistical value is displayed in the supporting information.

	$V_{OC}$ ± SD [V]	$j_{SC}$ ± SD [mA/cm$^2$]	$\eta$ ± SD [%]	FF ± SD [%]
Eva 1	0.90 ± 0.03	2.78 ± 1.01	1.22 ± 0.39	51.31 ± 7.63
Eva 2	0.94 ± 0.04	1.64 ± 0.69	0.52 ± 0.24	34.67 ± 2.18
Eva 3	0.96 ± 0.03	8.01 ± 0.90	4.31 ± 1.03	54.87 ± 9.29
Eva 4	1.02 ± 0.00	7.83 ± 1.24	5.86 ± 0.76	73.75 ± 2.05

The main reasoning for this is that the deposition parameters used in this work corresponded to the ones used in our prior publication on the regular solar cell structure, where indeed satisfying device efficiencies had been obtained. The underperformance of the devices presented in the current work is therefore not expected to be related to the absorber growth conditions per se, but must be somehow related to the variation of the substrate/contact layers.

However, in view of the comparable processing conditions we are able to draw several valuable and important conclusions taking into account our current and the previous work as will be discussed in the following.

## Discussion

Bækbo et al. showed that decomposition of MAI into smaller structures (mainly HI and CH$_3$NH$_2$) occurs when MAI is evaporated^13^. The adsorption kinetics were described by Kim et al. discussing the adsorption of PbI$_2$ and MAI on the substrate surface^21^. Both groups only measured a minor impact of the MAI flux on the quartz crystal balance. Therefore the suggestion of Kim et al. was made that the nucleation process consists of a seed layer of PbI$_2$ which is used as preferential bond for the MAI components. MAPI is then formed by diffusion processes of MAI through the seed PbI$_2$ layers. These diffusion processes were also described for PbCl$_2$ by Bækbo et al. and Chen et al. while the latter work showed a conversion of a 150nm PbCl$_2$ layer into MAPbCl$_3$^13,22^. For simultaneous evaporations, Parrott et al. showed that the growth starts with nucleation of small islands of 8nm height^23^. Heinze et al. showed that an initial crystallization of PbI$_2$ is possible during simultaneous evaporation depending on the targeted chamber pressure^10^.

Here, we observed that the pre/post-deposition of one of the two precursors in dual source co-evaporation of perovskite absorbers has a strong impact on the absorber properties and solar cell performance. Interestingly, PbI$_2$ seed layers (Eva 2) neither lead to better crystallization nor improved solar cell performance. The in situ XRD showed that the PbI$_2$ seed layers pre-deposited in Eva 2 were not fully converted to MAPI, but remained unreacted throughout the deposition. Therefore, a diffusion-driven formation of MAPI was, at least, not completed. Heinze et al. reported an increase of solar cell efficiency with the pre-evaporation of PbI$_2$ in the n-i-p structure, even with remaining PbI$_2$ XRD peaks^10^. We therefore conclude that the PbI$_2$ itself is not necessarily detrimental to the absorber, e.g. by introducing deep defects at the MAPI/PbI$_2$ interface. On the contrary, PbI$_2$ has been claimed to have a passivating effect in several works^24,25^. The detrimental effect of the PbI$_2$ seed layers in this work are therefore attributed to their location at the HTL side in the p-i-n structure. In fact, depositing PbI$_2$ at the ETL side of the device through post-deposition showed an improvement (EVA 4), similar to the seed layers in the n-i-p structure of Heinze et al^10^.

Furthermore, it was seen that only the processes with an MAI seed layer (Eva 3 and Eva 4) achieved an absorber structure with a distinct granular structure, and an improved solar cell performance. Muscarella et al. showed that the crystal plane orientation does not necessarily determine the electronic or optic properties, but Hsiao et al. assume a positive effect of larger grains because of less interfaces as recombination centres between the contacts^14,26^.

Ou et al. found that the electronic properties of MAPI highly depend on the MAI/PbI$_2$ ratio allowing different doping states from p-doped to n-doped which is caused by the placement of donator defects in the crystal lattice^27^. However, the electronic properties are not only influenced by the electrons and holes as charge carriers. Eames et al. showed the ionic migration of iodine and therefore effects on the band diagram in dependence of the stoichiometry to occur in MAPI^28^. The EDX measurements revealed that the films prepared during the course of this work were on average either nearly stoichiometric, or slightly PbI$_2$-rich. With reference to the work of Ou et al., this implies that the MAPI absorbers on average are intrinsic or n-doped to different extents.

**Figure 7:**
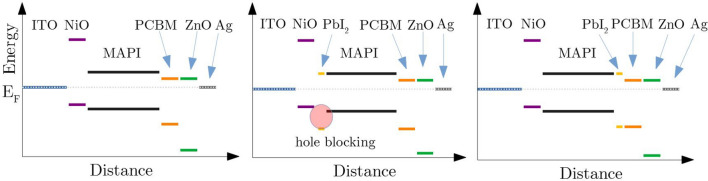
Schematic illustration of possible band diagrams including unconverted layers of lead iodide. The left image shows the solar cell with a fully converted MAPI absorber without residual PbI$_2$ (Eva 1). The middle image shows the solar cell with an additional layer of PbI$_2$ on top of the NiO (Eva 2). The right image shows the case of an unconverted lead iodide layer on top of the MAPI absorber (Eva 4).

**Figure 8:**
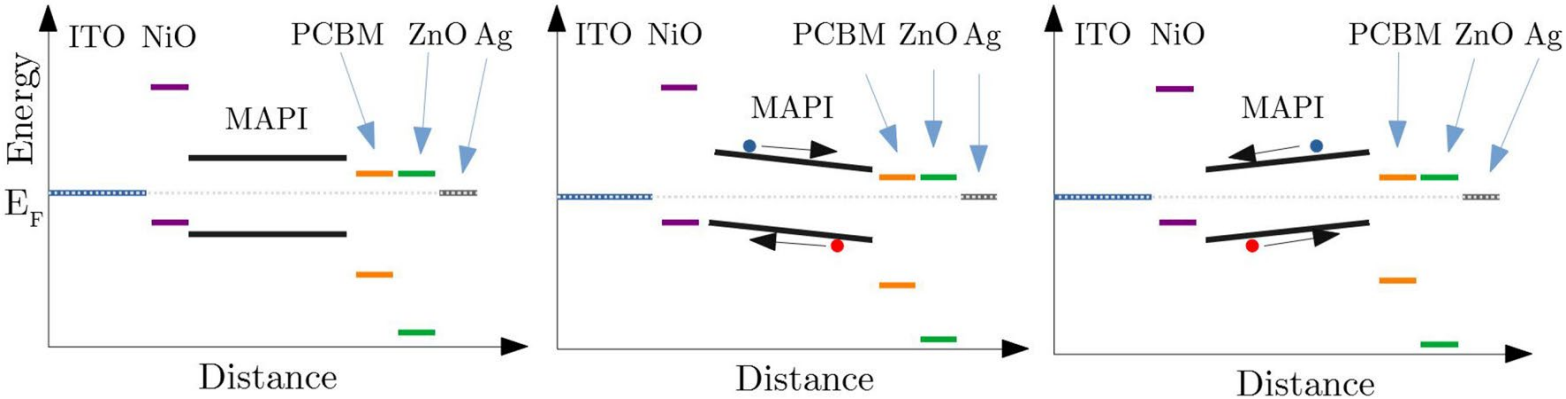
Schematic illustration of the band diagrams resulting from doping gradients within the MAPI absorber. Here, the blue circle illustrates the electrons and the red circle the holes. The left image shows the solar cell with a fully converted MAPI absorber without stoichiometry variations/gradients (Eva 1). The middle image shows a MAPI absorber with MAI-rich stoichiometry at the NiO interface and PbI$_2$-rich stoichiometry at the ETL interface (Eva 4). The right image shows a MAPI absorber whose stoichiometry is PbI$_2$-rich at the NiO interface and MAI rich on top of the absorber (Eva 2).

In order to explain the measured differences in solar cell performance, we present two hypothetical models as thought experiments. Figures 7 and 8 show illustrations of these possible effects of the different evaporation schemes on the electronic structure of our devices. The graphs present schematic sketches of possible band diagrams, where the placements of valence band, conduction band and Fermi level correspond to the literature data as measured by PES in references^24,29–33^.

According to these data from literature, due to the band offset in the valence band, an incomplete conversion of the PbI$_2$ layer covering the NiO (Eva 2) would lead to a hole blocking barrier as displayed in the band diagram presented in Fig. 7. Such an energy barrier for the holes traveling towards the HTL might drastically decrease the solar cell performance. Numerical calculations based on experimental data already showed the possibility and effect of such a band offset for a PbI$_2$/MAPI interface^34^.

If unconverted PbI$_2$ (Eva 4) is placed on top of the absorber, however, the effect would be opposite: Here, adjacent to the ETL, a blocking of the holes while letting the electrons pass through would actually support the filtering effect of the ETL.

TRPL measurements show that the decay times for high illumination intensities are reduced for the samples with PbI$_2$ post-deposition. One possible explanation is a larger density of trapping defects in the case of the pure MAPI layer or MAPI/PCBM interface, which would artificially prolong the observed decay time beyond the charge carrier lifetime through re-emission from the trap states^35^. A removal of the trap states at the interface by passivation with PbI$_2$ would in turn lead to reduced TRPL decay times, which is one possible interpretation of our TRPL data.

Golubev et al. numerically investigated the impact of buffer layers especially for a MAPI/C$_{60}$ interface. They calculated the influence of defect states on the open circuit voltage, which increases for a smaller number of these states^36^.

Furthermore, their numerical investigations were built on the experimental work of Liu et al., who reported a significant decrease in photoluminescence (PL) intensity and an increase in solar cell performance after placing a C$_{60}$ layer on top of a perovskite^37,38^. The numerical studies showed that this performance increase is probably a result of higher charge carrier mobility at the perovskite/C$_{60}$ interface, and therefore an improved charge transport across this interface^36^. This explains the decrease in PL by improved charge carrier extraction from the absorber, which leads to less radiative recombination and thus, lower PL intensity. The post-deposited PbI_2_ layer could act in a similar way as the C$_{60}$ buffer layer does, because the decay times at high intensities in our experiments were smaller as compared to the sample without post-deposition, while the open circuit voltage increased. These considerations would also match the research of Jacobsson et al., who studied the influence of remnant lead iodide on the MAPI absorber^24^. They found that lead iodide excess can improve the charge carrier extraction and leads to a quenching of the PL. This would be a second possible interpretation of the observed reduction of TRPL decay times in combination with an improved open circuit voltage. Additional measurements in this respect are needed to lead to more conclusive results.

A different thought model explaining the performance increase for the pre/post-deposition schemes is displayed in Fig. 8. As discussed before, the stoichiometry has a direct influence on the MAPI doping^27^. The n-i-p structure showed improved performance when PbI_2_ was pre-evaporated^10,13^. Even for fully converted PbI$_2$ layers, it could be expected that the first MAPI layers after PbI$_2$ pre-evaporation would possess a PbI$_2$-rich stoichiometry. In the case of the p-i-n structure this would lead to n-doped areas of MAPI close to the HTL^27,39,40^. The other way around, a pre-deposition of MAI would lead to a p-doped absorber interface adjacent to the HTL.

Following this line of thought, the pre- and post-evaporations induce stoichiometry gradients in the absorber, which in turn result in doping gradients and a band bending of the absorber at the interfaces. If the band bending fits the alignment of the ETL and HTL, the charge carrier separation and migration to the corresponding contacts is supported. Otherwise the charge extraction is impeded. This would also mean that even if the layers of pre-deposited PbI_2_ were fully converted to MAPI in the (inverted) p-i-n structure, this setup is not preferable due to the mismatching band bending. Transferring these considerations to the pre-evaporation of MAI and the post-evaporation of PbI_2_ (Eva 4), the absorber would be p-doped on the NiO interface (MAI-rich), intrinsic in the bulk (stoichiometric) and n-doped at the PCBM interface (PbI$_2$-rich), a configuration which would support charge extraction. These scenarios are depicted schematically in Fig. 8.

While we are unable to distinguish between the two hypotheses or confirm them further at this point, we present them here in order to inspire further thoughts along this line and would like to encourage additional research in this direction. It is clear, that both effects could also occur to different extends in parallel.

## Conclusion

This work showed that the pre-deposition of PbI$_2$ in the inverted p-i-n structure (Eva 2) is strongly detrimental for perovskite solar cell performance, in contrast to our previous results for solar cells in the regular n-i-p configuration. On the contrary, the pre-evaporation of MAI in combination with the post-evaporation of PbI$_2$ is beneficial (Eva 4) and showed the best solar cell efficiencies. While the pre-evaporation of MAI strongly improved the short circuit current density, the PbI$_2$ post-deposition mainly resulted in an increased fill factor and open circuit voltage.

Two thought models potentially explaining the influence of the sequential evaporations schemes on the measured performance have been portrayed. The first model presumes the evaporation of an unconverted PbI$_2$ layer to create an energy barrier for the holes in the p-i-n structure due to the mismatched band offset^24,29–33^. This would be beneficial at the ETL interface (post deposition of PbI$_2$), but not desirable at the HTL side. The second model assumes a conversion of pre- and post-evaporated layers, but with a remaining stoichiometry gradient, ultimately resulting in a doping profile within the absorber. By pre- and post-evaporation of MAI and/or PbI$_2$, it is possible to tune the doping of the MAPI at the top/bottom interface and therefore create a band bending which aids or hinders the charge carrier separation^13,21,27,39,40^. Both models are not in conflict with each other and in practice a combination of both effects is considered to be most likely.

For the first time different pre- and post-deposition sequences have been applied to MAPI solar cells in p-i-n configuration. Our results underline the importance to consider stoichiometry variations within the absorber and at the absorber interface during processing, especially in view of industrial in-line processing with non-stationary substrates. Together with our previous results, this work stresses the importance of initial and final steps of a co-evaporation process and sheds light onto the basic concepts of non-stationary processing schemes stimulating both exciting further scientific investigations and innovative technological processing options. We are confident that our results and lines of thought will enable and trigger a wide range of further research activities on dynamic and pre/post-evaporation schemes for co-evaporated solar cell absorbers.

## Supplementary Information

Supplementary Information 1.

## Data Availability

The datasets generated and analyzed during the current study are available from the corresponding author on reasonable request.
