# Supplementary material for: Impact of dynamic co-evaporation schemes on the growth of methylammonium lead iodide absorbers for inverted solar cells
Source: Sci Rep. 2022 Nov 10;12:19167. doi: 10.1038/s41598-022-23132-w (PMC9649758; doi:10.1038/s41598-022-23132-w)
Supplement: Supplementary file 1 — Supplementary Information 1. [file 41598_2022_23132_MOESM1_ESM.pdf]

Supporting Information

Impact of dynamic co-evaporation schemes on the growth of methylammonium lead iodide absorbers for inverted solar cells

Robert Heidrich [1,3], **Karl L. Heinze** [1,*], **Sebastian Berwig**[1], **Jie Ge**[1], **Roland Scheer**[1], **and Paul Pistor** [1,2,**]

[1]Martin-Luther-University, Von-Danckelmann-Platz 3, Halle (Saale), 06120, Germany
[2]Universidad Pablo de Olavide, c/ Utrera 1, Sevilla, 41013, Spain
[3]Fraunhofer CSP, Otto-Eißfeldt-Straße 12, Halle (Saale), 06120, Germany
[*]karl.heinze@physik.uni-halle.de
[**]ppis@upo.es

Experimental details - Evaporation chamber and chemicals

A glovebox filled with nitrogen is attached to the co-evaporation chamber which enables the crucible filling and sample exchange to be carried out in an inert gas atmosphere. The evaporation chamber allows a use of 5 evaporation sources in total, but in this work only 2 sources have been used for the evaporation of methylammonium iodide (MAI) and lead iodide (PbI_2). The substrate holder can fit up to 6 samples and is mounted directly on top of the evaporation sources. Flux measurements are possible with a quartz crystal microbalance (QCM) placed next to the substrate holder. The distance from the different crucibles to the sample which is placed in the centre of the substrate holder was measured to be 180 mm for the MAI source and 275 mm for the PbI_2 source.

As QCM an Inficon QCM IPN 074-156L was used in combination with an IQM-233 PN074-584-P1B Deposition Controller. Measured values were saved as csv data and evaluated in python. The derivative was calculated numerically applying a Savitzky-Golay filter on the raw frequency data. This was enabled with the help of the *scipy.signal* package in python to smooth the frequency values.

For pressure measurements an Edwards WRGS-NW 35 was used. This wide range sensor covers the required pressure interval from 1×10^{-6} mbar to 1×10^{3} mbar. Depending on the used evaporation rack, the data reading was either possible by using a LabVIEW program or by manual reading from the display.

Createc V-CRC-63-80-326-WK-S-M (serial number: EC017-1579) heaters have been used as evaporation sources. Crucibles were placed inside the heaters and the evaporation stream was controlled with shutters over both crucibles. For the temperature regulation Eurotherm 3500 and Eurotherm 2132 regulators have been used depending on the evaporation rack.

The two source materials used for the co-evaporation have been commercially purchased. (MAI (CAS No: 14965-49-2) from Ossila with a purity $> 99.9\,\%$, product code: M271. PbI_2 (CAS No: 10101-63-0) from VWR (supplier Alfa Aesar) with a purity $\geq 99.9985\,\%$, product code: 12724.)

Experimental details - Characterisation methods

j-V characterisation

The solar cell j-V characterisations were done in a standardized setup using a sun simulator with halogen lamp in combination with a Keithley 2400 source measure unit. To contact the substrate, a substrate holder with lead-outs enables a 2-wire measurement. The active area was limited to $0.096\,\mathrm{cm}^2$ for every solar cell by applying a shadow mask. For measuring the current-voltage characteristics, the setup is calibrated with a silicon cell with known parameters to an illumination of one sun ($100\,\mathrm{mW/cm}^2$, 25 °C, AM 1,5). All measurement settings except lamp intensity and position are controlled by a home-made LabVIEW program. After selecting the desired adjustments, the program independently measures the values and saves them into a *.txt* file.

SEM and EDX measurements

The used scanning electron microscope (SEM), was a Zeiss Supra 40 VP, which is based on the GEMINI technology. Mainly the Inlens detector was used with working distances from 1.5 mm to 3 mm. The applied voltage was fixed at 3 kV with the

20 µm aperture. Magnification values between 5000 and 100000 were used.

For the energy dispersive X-ray spectroscopy (EDX) measurements a Bruker XFlash 630M was used. This detector was mounted on the Zeiss Supra 40 VP allowing EDX measurements directly after taking SEM images of the morphology. For this purpose, the magnification was fixed at 500 with 8 mm working distance. The used voltage was fixed at 10 kV with the 120 µm aperture. By using these parameters, the interaction depth reached values around 500 nm to 600 nm which secures a detection of all used layers. The data was evaluated with the help of the software ESPRIT 2.

In situ XRD measurements

The in situ X-ray diffraction (XRD) measurements were carried out through exchangeable Kapton windows in the chamber walls. Cu-K_α radiation at 35 kV and 40 mA with 1.54 Å wavelength was used for the measurements, and the K_β radiation was damped by a Ni filter to 5 % of the K_α intensity. For the XRD detection, three Dectris Mythen 1K detectors are connected to each other in a circular arc in front of the exit Kapton window. The used incidence angle of the X-Ray source was set at 11° allowing an in situ 2Θ range from 8° to 36°. Due to the connection points of the three Dectris Mythen 1k detectors, blind spots at 17.3° and 26.7° occur in the corresponding color maps.

TRPL analysis

Time resolved photo luminescence (TRPL) measurements took place in a standardized home-made single photon counting setup. A pulsed diode laser with a wavelength of 638 nm was used for creating charge carriers. The laser intensity was damped between 0.001 % and 100 % intensity with the help of a neutral density filter wheel. While the frequency of the laser pulse is in principle adjustable, for these experiments a frequency of 1 MHz was used. The width of every laser pulse is 88 ps with 12.6 pJ energy and a focus area of 4.5×10^{-5} cm^2 creating an energy density of 267 nJ/cm^2 in the full intensity modality. A "Pico Quant" detector was used for the photon detection with wavelengths between 270 nm and 850 nm. The time interval for every channel of the multi-channel analyser was set to 56 ps which is therefore the time increment of the data points. The measurement of higher excitation laser intensities is coupled to a higher recombination rate. To avoid immediately reaching the counting limit of the detector, damping filters in front of the detector were placed for several measurements. These filters can attenuate the incoming photon rate from the laser between 1 % and 100 % before reaching the detector. All measurements were saved into a *.txt* file for evaluations in python.

Used substrate basis and preparation of the solar cells

ITO layer and cleaning process

For all processes ITO coated glass substrate were used. These samples were purchased from Kintec with the pattern KT18274 and 25.0 mm × 25.0 mm × 1.1 mm size. The ITO surface showed a polished grade with a conductivity of 10 Ω/sq. Every cleaning and NiO preparation run was consisting of 24 substrates allowing different process variations for the same substrate batch. After removing the substrates from the package, every sample was engraved with a sequence number starting from R000. When the engraving was finished, the substrates were placed inside probe carriers filled with isopropanol. These probe carriers were put into an ultrasonic bath for 25 min at 50 °C. After the ultra sonic bath, every substrate was removed from the probe carrier, washed up with distilled water and dried with a nitrogen stream.

Synthesis of the NiO layer

The NiO layer was deposited via electron beam evaporation in an *Alliance Concept Eva*450 chamber. The substrate carrier fits all 24 prepared samples and was rotating while the deposition was running. Furthermore, the substrate carrier design allows to place all samples at approximately the same distance from the substrate carrier centre. Due to these facts, there should only be minor difference of the NiO layer deposition between the different samples depending on geometric factors. All homemade NiO layers were fabricated at 150 °C and 8 sccm O_2 for 4 min which led to a layer thickness of approximately 20 nm to 30 nm.

Synthesis of MAPI solar cells

When the absorber was finished, the buffer layer PCBM had to be deposited. This film was created with a 20 $\frac{\text{mg}}{\text{ml}}$ solution in chlorobenzene. The solution was dropped onto the substrate, which was rotated at 2000 rpm for 50 s. After the buffer layer, the ETL, consisting of ZnO nano particles in a dispersion of 2.5 wt% in isopropyl alcohol (IPA), was spin-coated at 4000 rpm for 30 s with a subsequent heating at 90 °C for 5 min. The last step is the evaporation of the metal electrodes which was realized by evaporating 100 nm thick Ag-films with the help of a shadow mask.

Evaporation schemes

The subsequent passage visualizes the temperature and pressure development of all evaporation schemes in Figure 1. Blue curves indicate the PbI_2 temperature, green curves the MAI temperature, red curves the substrate temperature and black points

the measured pressure. Dashed lines inside the plots mark the opening and closing times of the corresponding shutter. The following acronyms were used in the submitted work:

- Eva 1: simultaneous evaporation of MAI and PbI_2
- Eva 2: pre-evaporation of PbI_2
- Eva 3: pre-evaporation of MAI
- Eva 4: pre-evaporation of MAI and post evaporation of PbI_2

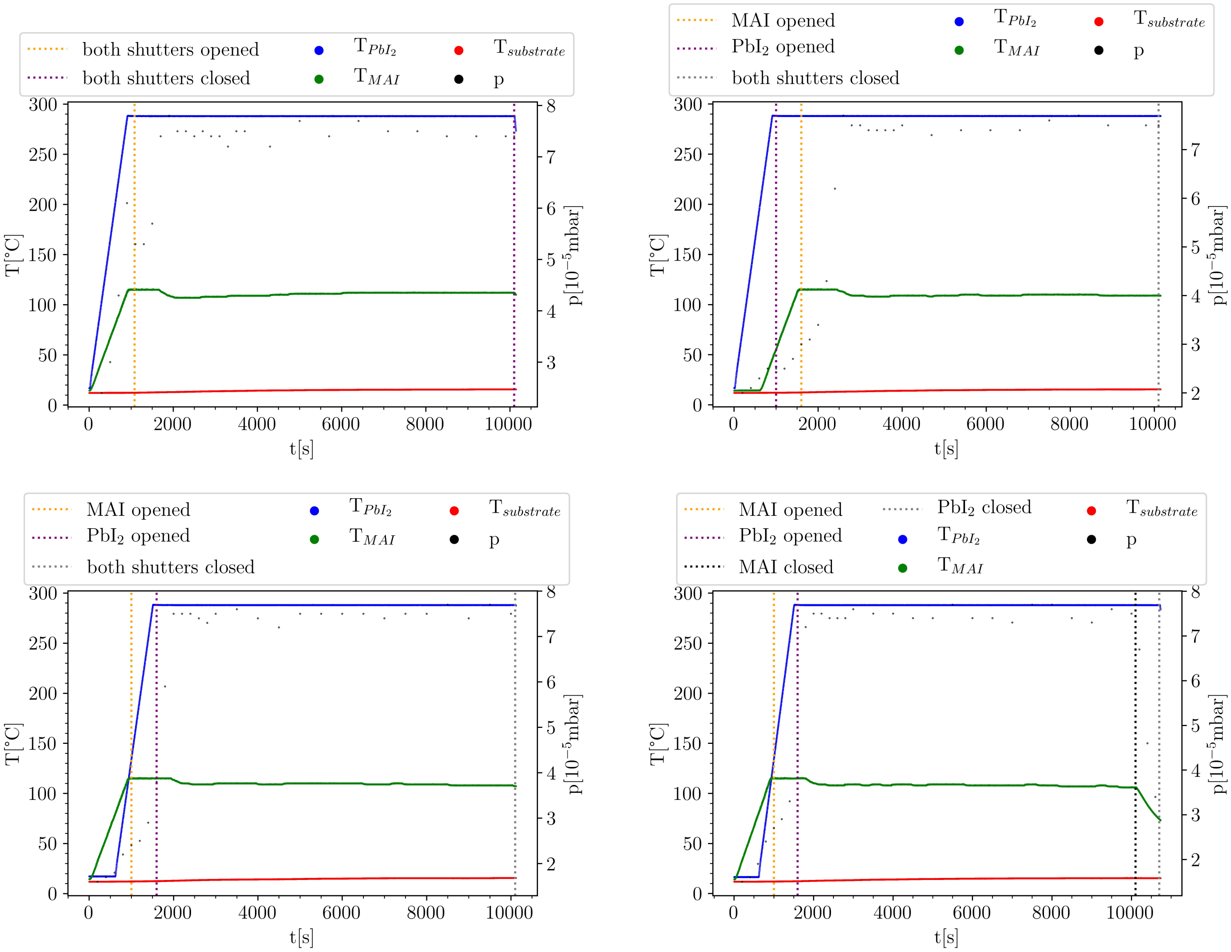

Figure 1. Development of the evaporation parameters. Eva 1 (top left), Eva 2 (top right), Eva 3 (bottom left), Eva 4 (bottom right).

Figure 2 displays the corresponding frequency development of the QCM. The black curves indicates the raw frequency data while the green curve shows the time derivative of the frequency. As it was shown in the previous plots, the dashed lines are marking the opening and closing times of the shutters. Especially the results of pre- and post-evaporations and the impact of shutters can be observed in the time derivative of the frequency. Furthermore, a constant time derivative is indicating a stable deposition process.

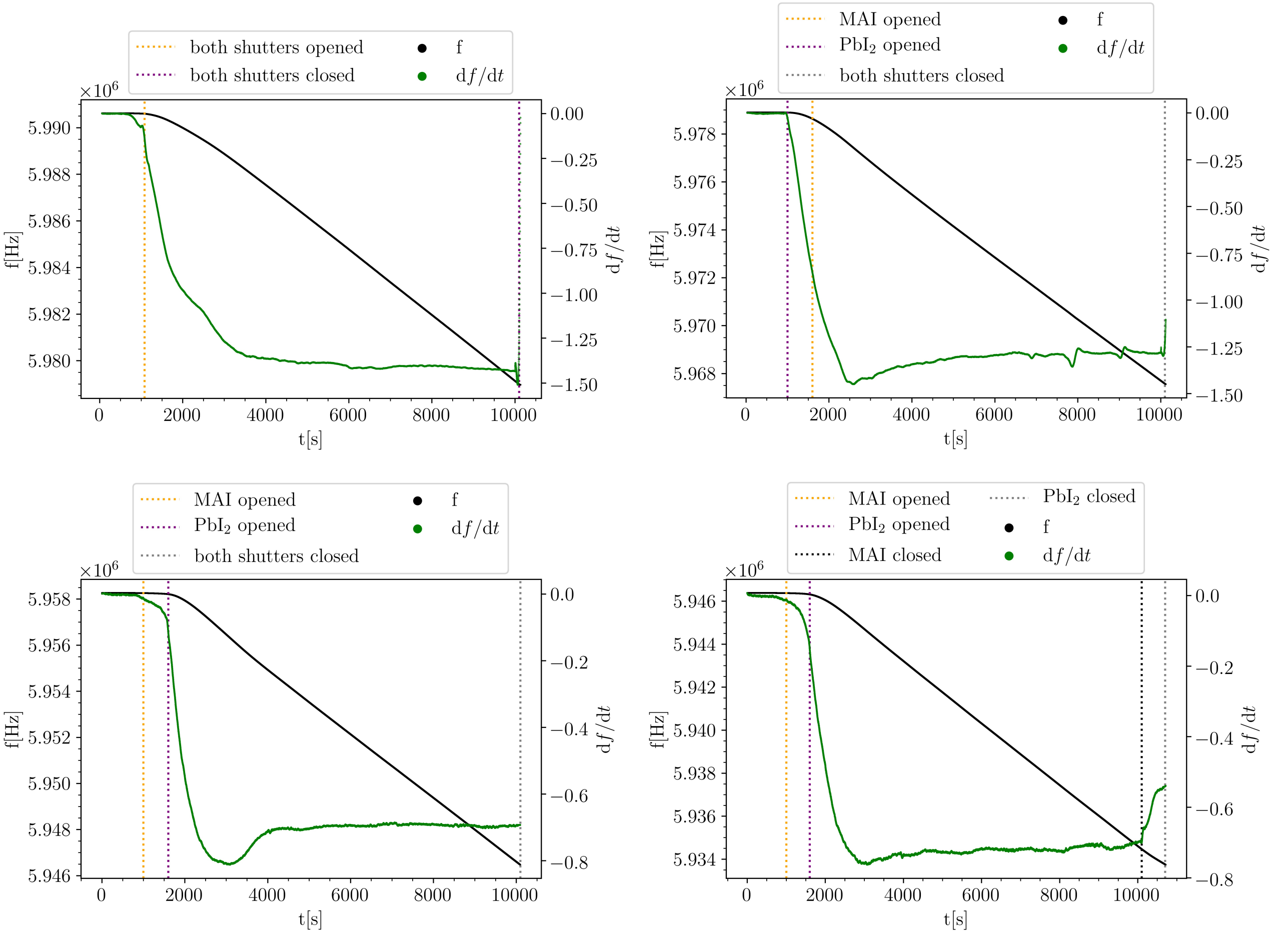

Figure 2. Development of the frequency data. Eva 1 (top left), Eva 2 (top right), Eva 3 (bottom left), Eva 4 (bottom right).

j-V parameter statistics

During the experiments a large quantity of solar cells were prepared. Figure 3 and Figure 4 list the key characterization parameters of the j-V analysis for Eva 1-4. The box-plot visualizes the statistic distribution while the orange line marks the mean values. In addition, all measured values are presented as circles next to the box plot. Non-functional cells did not contribute to the statistics but were mentioned in the corresponding figure caption.

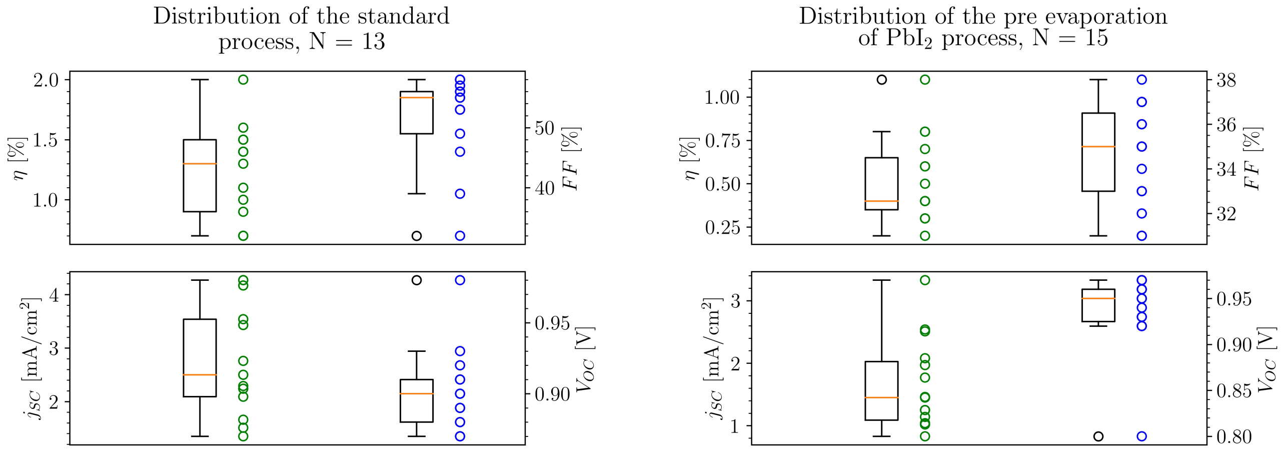

Figure 3. Left: Performance of the solar cells manufactured via Eva 1, non-functional cells: 2, number of batches: 2. Right: Performance of the solar cells manufactured via Eva 2, non-functional cells: 0, number of batches: 2.

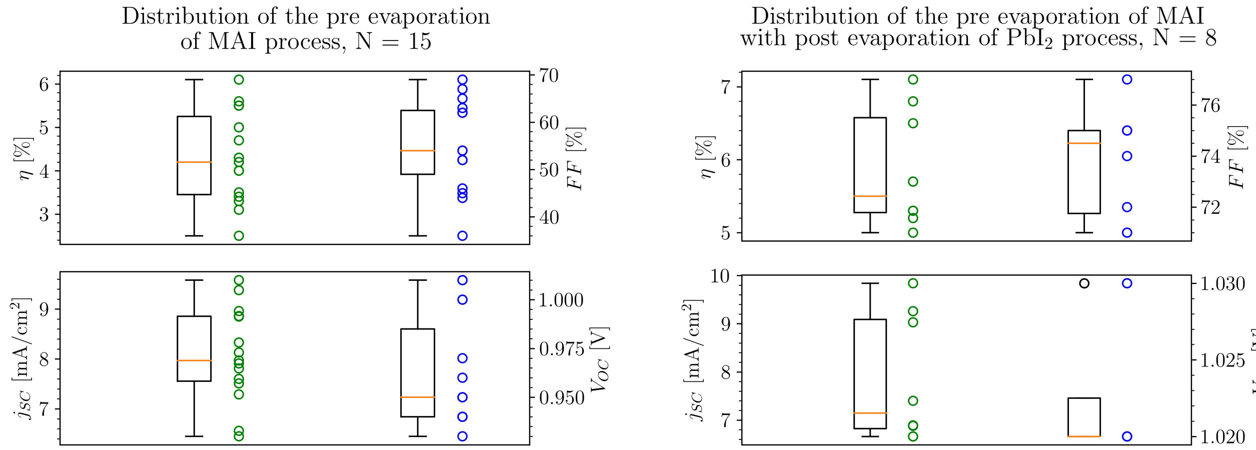

Figure 4. Left: Performance of the solar cells manufactured via Eva 3, non-functional cells: 0, number of batches: 2. Right: Eva 4, non-functional cells: 1, number of batches: 1.

Morphology absorber images

The absorber morphology in dependence of the corresponding evaporation scheme is displayed in Figure 5. With respect to the EDX measurements, larger grain structures seem to require a $\frac{I}{Pb}$ stoichiometry of 3 or above. However, the cross section images of EVA 4 also suggest large grains with a top layer of smaller grains probably caused by the PbI_2 post depostion. Therefore, it can be concluded that the pre-deposition of MAI on NiO is beneficial for the crystallization behavior of MAPI in the inverted structure.

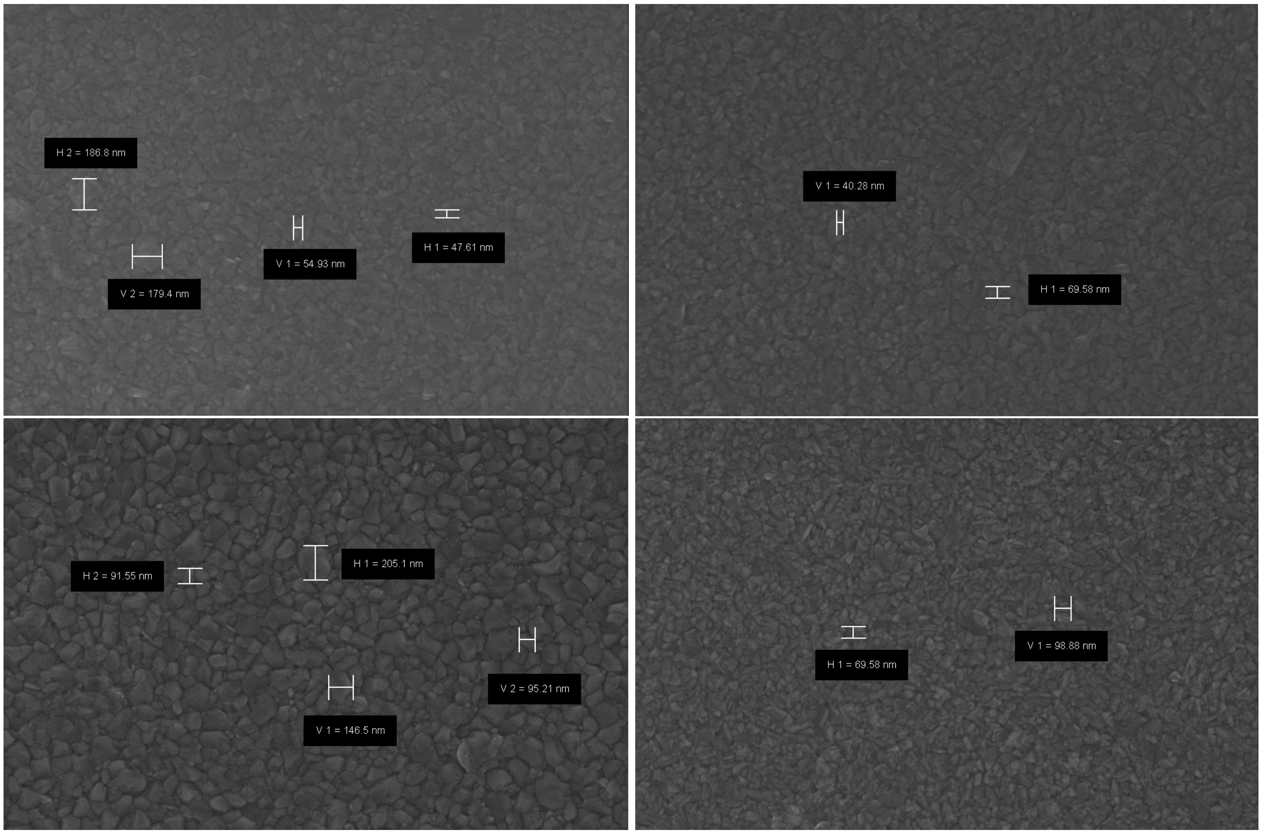

Figure 5. Morphology SEM images of the perovskite absorbers of Eva 1 (top left), Eva 2 (top right), Eva 3 (bottom left) and Eva 4 (bottom right). The magnification was fixed at 80000.

Statistics of the wet chemical reference cells

Wet chemical reference cells were fabricated from the same substrate batch as the coe-evaporated samples presented in the main manuscript. The base substrate (glass and ITO) and the NiO deposition are equal to the preparation scheme for the co-evaporated solar cells. The absorber was prepared as follows:

The source solution for the wet chemical MAPI deposition consists of 658 mg PbI_2 and 228 mg MAI, which were dissolved in 1 ml co-solution containing 100 µl dimethyl sulfoxide (DMSO) and 900 µl dimethyl formamide (DMF). The absorber layer was spin-coated by dropping 100 µl of the source solution on an ITO/HTL substrate and spinning at 4000 rpm for 30 s. While the substrate was rotating, 1 ml diethyl ether was given onto the substrate after 10 s. Afterwards, the substrate was heated at 100 °C for 10 min. All following steps are equal to the manufacturing of the co-evaporated solar cells.

Figure 6 and Table 1 are listing the key parameters of the wet chemical reference cells. The summary of all wet chemical reference samples shows a large variation of solar cell parameters. While efficiencies over 15 % have been achieved, the average efficiency is only 9.57 % due to the large distribution of measurement values. Especially the comparison with the co-evaporated absorbers is of great interest. Also, the wet chemical reference samples show not only a large distribution of key parameters, but 2 of 6 cells are non-functional, indicating a problem which is not necessarily caused by the absorber. While the efficiency of the wet chemical reference process is in general higher due to higher current densities, the fill factor is heavily increased by EVA 4. However, the statistical distribution shows that the base process for both preparation methods is not optimized at this state of work.

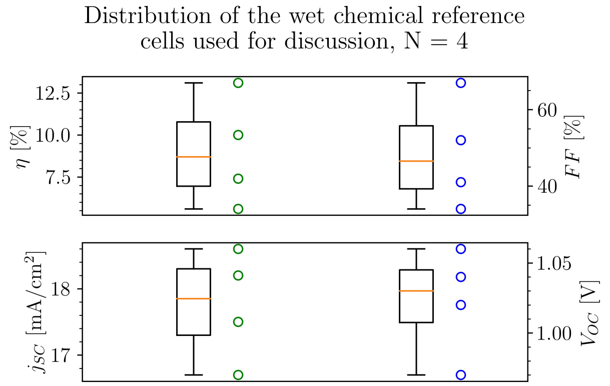

Figure 6. Performance of the solar cells manufactured with a wet chemical absorber process from the same batch as the co-evaporated solar cells in the discussion, non-functional cells: 2, number of batches: 1.

	$V_{OC} \pm$ SD [V]	$j_{SC} \pm$ SD [mA/cm^2]	$\eta \pm$ SD [%]	FF $\pm$ SD [%]
all reference cells	0.99 ± 0.03	18.74 ± 3.03	9.57 ± 4.13	50.09 ± 16.84
reference cells of the discussed batch	1.02 ± 0.03	17.75 ± 0.72	9.03 ± 2.83	48.50 ± 12.46

Table 1. Average and standard deviation (SD) of solar cell parameters from wet chemical reference cells. The first line refers to the values of reference cells from 5 different batches of substrates (23 functional cells, 4 non-functional cells, number of batches: 5). The second line refers to wet-chemically prepared solar cells from the same substrate batch as the co-evaporated solar cells discussed in the main manuscript (4 functional cells, 2 non-functional cells: number of batches: 1)
